# Enterovirus Infections in Solid Organ Transplant Recipients: a Clinical Comparison from a Regional University Hospital in the Netherlands

**DOI:** 10.1128/spectrum.02215-21

**Published:** 2022-02-09

**Authors:** Hayley Cassidy, Coretta van Leer-Buter, Hubert G. M. Niesters

**Affiliations:** a Department of Medical Microbiology and Infection Prevention, Division of Clinical Virology, University of Groningen, University Medical Center Groningen, Groningen, the Netherlands; Johns Hopkins Hospital

**Keywords:** enterovirus infection, transplantation, immunocompromised, pediatric infectious disease, viral infections, enterovirus D68

## Abstract

Enterovirus infections are known to cause a diverse range of illnesses, even in healthy individuals. However, information detailing enterovirus infections and their severity in immunocompromised patients, such as transplant recipients, is limited. We compared enterovirus infections in terms of genotypes, clinical presentation, and severity between transplant and nontransplant patients. A total of 264 patients (38 transplant recipients) with 283 enterovirus infection episodes were identified in our hospital between 2014 and 2018. We explored the following factors associated with enterovirus infections: clinical presentation and diagnosis on discharge, length of hospital stay, symptom persistence, and infection episodes in both children and adults. We observed some differences in genotypes between patients, with enterovirus group C occurring mainly in transplant recipients (*P* < 0.05). EV-associated gastrointestinal infections were more common in patients with a transplant (children [71%] and adults [46%]), compared to nontransplant patients (*P* < 0.05). Additionally, nontransplant patients had a higher number of hospital stays (*P* < 0.05), potentially reflecting more severe disease. However, transplant patients were more likely to have symptom persistence after discharge (*P* < 0.05). Finally, children and adults with a transplant were more likely to have additional enterovirus infection episodes (*P* < 0.05). In our cohort, enterovirus infections did not seem to be more severe after transplantation; however, patients tended to present with different clinical symptoms and had genotypes rarely found in nontransplant recipients.

**IMPORTANCE** Despite the high prevalence of enteroviruses in the community and the increasing demand for transplants from an aging population, knowledge on enteroviruses in solid organ transplant recipients is currently limited. Transplant recipients represent a significant patient population and require additional considerations in patient management, particularly as they have an increased risk of disease severity. Enteroviruses are known to cause significant morbidity, with a diverse range of clinical presentation from over 100 different genotypes. In this study, we aimed to provide a more comprehensive overview of enteroviral infections in transplant recipients, compared to nontransplant patients, and to bridge some gaps in our current knowledge. Identifying potential clinical manifestation patterns can help improve patient management following enterovirus infections.

## INTRODUCTION

In recent years, improved antirejection treatment for solid organ transplantation has improved the outcome for transplant recipients (1). However, the suppression of the immune system, which is necessary for graft function, also leads to a greater susceptibility to infections. Viral infections are well recognized complications of immune suppression and can occur through reactivation of latent viruses (2).

Enteroviruses are ubiquitous in the community, with studies indicating an 11–13% prevalence even among healthy individuals (3, 4). In the majority of patients (90%), most enterovirus infections are asymptomatic or present with mild or indiscriminate respiratory or gastrointestinal symptoms (5). Some of these clinical syndromes are mostly associated with one or few enterovirus genotypes, while others are typical for a particular patient group, such as coxsackievirus B3 and neonatal sepsis (6). However, in an immunocompromised population, a simple gastroenteritis can have large consequences. These patients are more likely to have severe manifestations with longer hospital admissions, exposing them to other hospital acquired infections and longer recovery (7). Moreover, symptoms of infections often resemble rejection, one of the most important complications in transplant patients. As the treatment of rejection results in an increased immunocompromised condition, it is extremely important that symptoms compatible with infection are diagnosed accurately. Nevertheless, it is equally important that symptoms are not falsely attributed to a bystander, delaying the appropriate management of a vulnerable patient.

Enteroviruses have vast variations in their genomes and can be categorized into four different family groups, A, B, C and D, each with their own subspecies, resulting in over 100 different genotypes (8). Each genome is comprised of a single polyprotein, flanked by 5′ and 3′ untranslated regions, and encodes four structural proteins (viral proteins 1–4) and seven nonstructural proteins (2A-2C and 3A-3D). Detection and confirmation of an enterovirus usually involves targeting the 5′ UTR during real-time reverse transcriptase PCR. To distinguish between genotypes, sequencing of the VP1 gene present on the viral capsid remains the gold standard (9).

Enterovirus infections following transplantation are neither fully understood nor studied. Indeed, literature on enterovirus infections in transplant recipients consists mainly of case studies and small patient cohorts, involving either one type of organ transplant or specific enterovirus genotypes (10, 11). It has been revealed that different enteroviruses can target different cell receptors (4). While some are thought to cause direct damage from replication, such as enterovirus D68 (EV-D68), others appear to cause damage from secondary host inflammatory responses, such as coxsackievirus B4 during myocarditis (12). Therefore, it could be reasoned that immunocompromised individuals or age groups could have a different clinical presentation or outcome following infection with different enterovirus genotypes. Identification of the genotype is important to be able to track trends in outbreak situations and link specific clinical presentation. As a result, continued surveillance and reporting of enterovirus infections is important, not only for diagnostics and assay development, but also for patient management.

A more comprehensive overview of enterovirus infections in solid organ transplant recipients, both in children and adults, may be required to bridge some gaps in our current knowledge. In this study, we compared transplant patients with nontransplant patients by (i) investigating the distribution of genotypes, (ii) exploring clinical manifestations, and (iii) determining potential differences in severity.

## RESULTS

### Description of patients with enterovirus detection.

**FIG 1:**
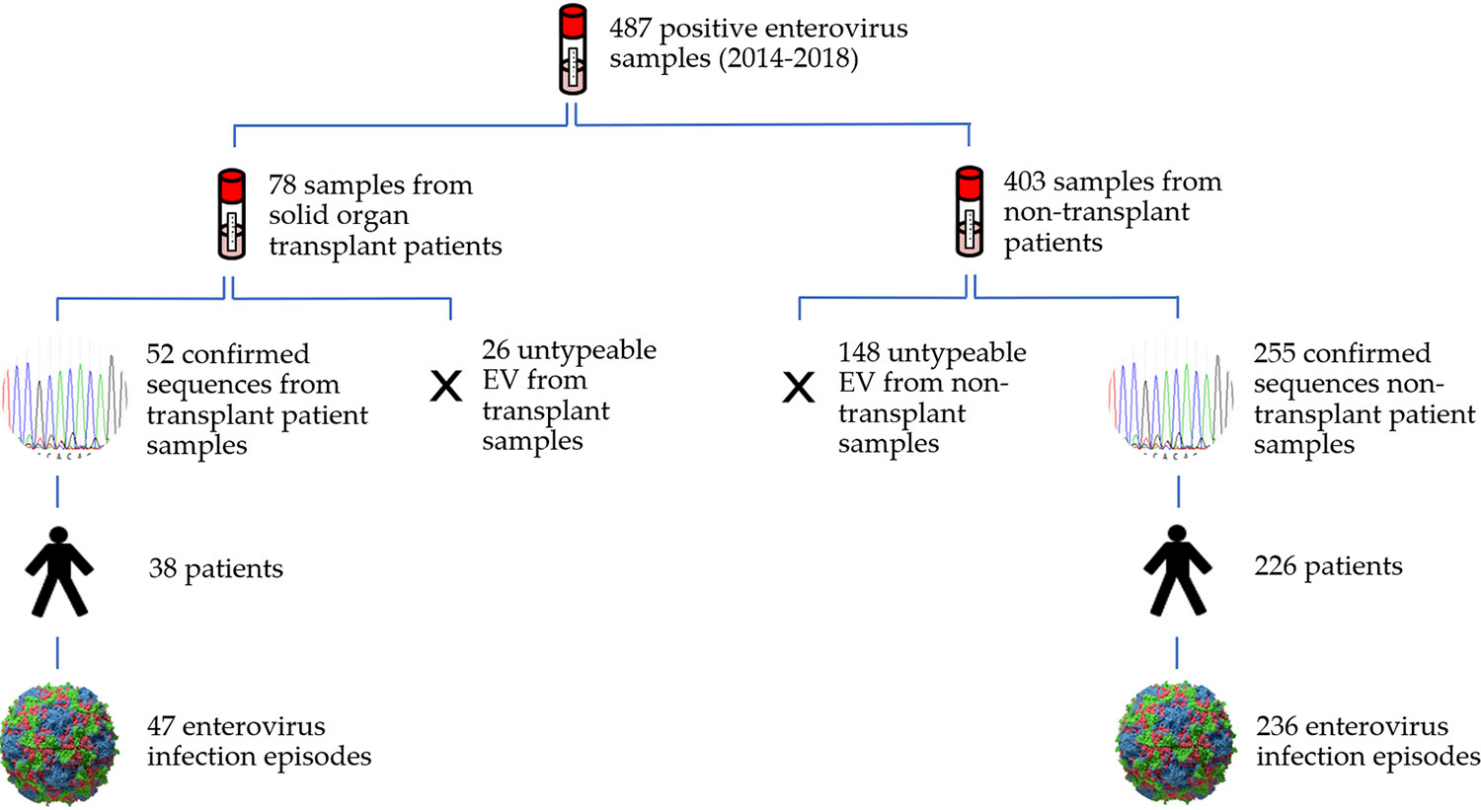
Overview of sample selection. EV, enterovirus. The license for the enterovirus particle illustration: CC BY SA 3.0.

A total of 264 patients with 283 enterovirus infection episodes, covering 33 genotypes, were included in the study. Overall, 23 transplant recipients (26 samples) and 128 nontransplant recipients (148 samples) were excluded due to a poor sequencing result (Fig. 1). For the purpose of analysis and comparison, enterovirus genotypes were divided into their corresponding family groups: EV-A, EV-B, EV-C, or EV-D (Tables 1, 2 and S1) (8). The following tables depict the general patient and sample characteristics of each population (Tables 1 and 2).

**TABLE 1:** Clinical characteristics of patients without a transplant

Clinical and sample characteristics			Nontransplant recipients			
			Children		Adults	
			0-5 yrs	6-15 yrs	>15 yrs	Total
Patients	Gender	Male	65	30	29	124
		Female	44	23	35	102
		Total no.	109	53	64	226
Comorbidities	Category	Pulmonary	9	15	8	32
		Cardiac	11	2	6	19
		Renal	0	1	1	2
		Abdominal	13	7	6	26
		Serve disability	9	12	2	23
		Malignancy	3	4	8	15
		Prematurity	22	3	1	26
		Diabetes	0	1	4	5
		Immune deficiency	3	1	5	9
		Neurological	6	2	3	11
		Total no.	67	48	44	168
Samples	Type*^1^^*a*^	Fecal	65	19	11	95
		Respiratory	40	34	28	102
		Cerebrospinal fluid*^3^	16	2	19	37
		Blister fluid	2	0	5	7
		Other*^4^	3	1	3	7
		Total no.	126	56	66	248
Enterovirus detection	Detected family group*^2^	EV-A^*b*^	41	15	9	65
		EV-B	55	16	23	94
		EV-C	3	4	13	20
		EV-D	17	21	19	57
		Total no.	116	56	64	236

a *1, Duplicate sample materials from the same infection were removed. *2, Duplicate detections from the same infection were removed. *3, It must be noted that for several of the patients, the enterovirus viral load in some of the CSF samples were too low for typing, and as a result, an alternative sample material had to be taken. *4, Other sample types consisted of plasma and heart tissue.

b EV-A, enterovirus group A. EV-B, enterovirus group B. EV-C, enterovirus group C. EV-D, enterovirus group D.

**TABLE 2:** Clinical characteristics of transplant patients

Clinical and sample characteristics			Transplant recipients			
			Children		Adults	
			0-5 yrs	6-15 yrs	>15 yrs	Total
Patients	Gender	Male Female	2	3	15	20
			3	4	11	18
		Total no.	5	7	26	38
	Transplant	Lung	0	0	12	12
		Liver	5	6	3	14
		Kidney	0	1	8	9
		Heart	0	0	2	2
		Multiorgan*^1^^*a*^	0	0	1	1
		Total no.	5	7	26	38
Sample	Type*^2^	Fecal	7	8	16	31
		Respiratory	0	1	15	16
		Cerebrospinal fluid	0	0	0	0
		Blister fluid	0	0	1	1
		Other*^4^	0	1	0	1
		Total no.	7	10	32	49
Enterovirus detection	Detected family group*^3^	EV-A^*b*^	3	6	2	11
		EV-B	4	2	0	6
		EV-C	0	2	17	19
		EV-D	0	0	11	11
		Total no.	7	10	30	47

a *1, One adult patient had a lung and liver transplant. *2, Duplicate sample materials from the same infection were removed. *3, Duplicate detections from the same infection were removed. *4, Other sample types consisted of plasma and heart tissue.

b EV-A, enterovirus group A. EV-B, enterovirus group B. EV-C, enterovirus group C. EV-D, enterovirus group D. A multinomial logistic regression analysis revealed the odds of having a group C (OR 15.67; 95% CI 5.58, 43.98; *P* < 0.05) and group D detection (OR 4.40; 95% CI 1.63, 11.89; *P* < 0.05), rather than a group B detection was statistically higher in transplant patients, compared to nontransplant patients.

**(i) Patient characteristics.** Most patients included in our study were children younger than 16 years (174 children versus 90 adults), with an average age of 5 years in children and 45 years in adults (Tables 1 and 2). While the most frequent underlying condition in small children (<6 years) without a transplant was prematurity, it was pulmonary disease and severe disability in older children (6–15 years). Adults without a transplant, compared to children, had more varied underlying conditions, with malignancy and pulmonary disease (including five cases of asthma) being the most frequent comorbidity. Of the adults without a transplant, only 23 (35.9%) were previously healthy, underscoring the tertiary care function of this hospital. Transplant recipients formed 14.4% of patients, with the majority being adults. Indeed, only 12 patients (average 6 years) under the age of 16 were transplant recipients (Table 2). In small children, only liver transplants (*n* = 5) were identified and in older children, six liver and one kidney transplant recipients were included. Meanwhile, in adults, lung transplant recipients (*n* = 12, 46.2%) represented the largest group, followed by kidney transplant recipients (*n* = 8, 30.8%). No significant differences were found between males and females (*P* = 0.79).

**(ii) Sample characteristics.** In small children, enteroviruses were most frequently found in feces (Tables 1 and 2). However, in older children without a transplant, enteroviruses had a significantly higher detection in respiratory material, compared to transplant patients of this age group (*P* = 0.009). In adults, enteroviruses were most commonly detected in fecal samples from transplant recipients and respiratory samples from nontransplant recipients, with 63.3% and 42.4%, respectively. In comparison, only 16.7% of enteroviruses were detected in feces in nontransplant adults. Interestingly, of the 16 respiratory samples collected from transplant patients, 10 detections were from lung transplant recipients. Most strikingly, enteroviruses were only detected in cerebrospinal fluid (CSF) from nontransplant patients.

### Enterovirus detection and genotype distribution.

Although EV-A and EV-B were most commonly detected in children in both populations, EV-D68 was the most frequently identified genotype in children without a transplant (Table S2). Meanwhile echovirus 11 (*n* = 3, 17.5%) was the most frequently detected genotype in children with a transplant and occurred solely in liver transplant recipients (Table S2). Interestingly, EV-C were most commonly detected in adults with a transplant, comprising of 57% (*n* = 17) of detections. Coxsackievirus A22 (CV-A22) was subsequently identified as the most frequently detected EV-C genotype in transplanted adults (*n* = 8, 26.7%), occurring in all solid organ transplants included in the study (Table S2 and S3). In comparison, there were only 13 EV-C detections, including one CV-A22 detection, in adults without a transplant (20% of adult detections). EV-D (in which the only genotype detected was EV-D68) were found in all ages in the nontransplant population and exclusively in adults in the transplant population (Tables 1 and 2). EV-C and EV-D detections occurred significantly more in transplanted individuals than in nontransplanted individuals, compared to EV-B detections (*P* < 0.05). A complete breakdown of the number of genotypes found in each sample material and transplant type can be found in Tables S2, S3 and S4.

**(i) Co-detections.** An enterovirus was the only pathogen identified in 21.3% (*n* = 10) of detections in transplant patients and 46.6% (*n* = 110) of detections in nontransplant patients. Rhinoviruses were found to be the most commonly identified co-detection (29.85%) associated with infections in the nontransplant population. Furthermore, co-detections with adenovirus (10.6%), along with other viruses associated with gastroenteritis, such as rotavirus (4.3%) and norovirus (4.3%), were additionally found in the nontransplant population. Similarly, co-detections with adenovirus were observed in the transplant population, along with norovirus (20%) and human parainfluenza type 3 (13.3%). A detailed overview of the co-detections found at the same time as the detected enterovirus are shown in Tables S5 and S6.

### Clinical manifestations from enterovirus infections.

Based on the documented clinical symptoms at presentation, patients were divided into four categories (usually directed by the type of clinical sample taken from the patient upon admission): respiratory, gastroenteritis, neurological or other (including HFMD, myocarditis, and sepsis) (Table 3). As age is known to be a significant factor in determining the disease prognosis, we also explored differences between children (<16 years) and adults. Only enteroviruses which were deemed to be the causative agent of the clinical manifestations were investigated (Table S7).

**TABLE 3:** Clinical manifestations attributed to the detected enterovirus

Clinical manifestations*^1^^*a*^			Nontransplant recipients		Transplant recipients	
			Children (*n* = 74)	Adults (*n* = 43)	Children (*n* = 6)	Adults (*n* = 11)
Enterovirus infections	Clinical presentation	Respiratory*^2^	37 (47%)	10 (23%)	2 (29%)	5 (38%)
		Gastrointestinal*^3^	12 (15%)	1 (2%)	3 (43%)	5 (38%)
		Neurological*^4^	8 (10%)	25 (58%)	0	2 (15%)
		Other*^5^	21 (27%)	7 (16%)	2 (29%)	1 (8%)
	Final diagnosison discharge	Respiratory infection	30 (38%)	11 (26%)	2 (29%)	6 (46%)
		Gastroenteritis	10 (13%)	3 (7%)	5 (71%)	6 (46%)
		Neurological infection*^6^	28 (36%)	21 (49%)	0	0
		Other infections*^7^	10 (13%)	8 (19%)	0	1 (8%)
		Total no.	78	43	7	13

a *1, Percentages were calculated from the total number of causative enterovirus infections for clinical presentation and final diagnosis on discharge. *2, Respiratory symptoms consisted of one or more of the following: cough, sore throat, breathing difficulties, cold or chills. *3, Gastrointestinal symptoms consisted of one or more of the following: diarrhea, abdominal pain or vomiting. *4, Neurological symptoms consisted of one or more of the following: headache, neck pain/stiffness, photophobia, or convulsions. *5, Other presentation consisted of vesicular rash, particularly on the hands and feet, cardiogenic shock, febrile illness, and impaired functions during sepsis. *6, One enterovirus, associated with a neurological infection, was not included in Table 3 as it was part of a coinfection with human parechovirus 3 (HpeV-3). *7, Hand Foot and Mouth disease, myocarditis, sepsis, and fibril illness.

**(i) Clinical manifestations in children.** Respiratory symptoms and subsequent respiratory infections were most commonly associated with enteroviruses detected in children without a transplant, with EV-D68 causing the majority of infections (*n* = 24, 80%) (Tables 3 and S8). Indeed, EV-D68 accounted for the majority of respiratory symptoms and infections both in patients without a transplant and in adults with a transplant in this study (*P* < 0.05). Interestingly, other than EV-D68, no other genotype was found to have caused more than one respiratory infection each, in the nontransplant population. In comparison, gastrointestinal symptoms (judged as an enterovirus gastrointestinal infection) were most commonly found in children with a transplant (*P* < 0.05) (*n* = 5 out of a total of 7 infections), of which three infections were EV-A, one EV-B and one EV-C (Table S8).

**(ii) Clinical manifestations in adults.** Notably, enteroviruses in adults without a transplant were most frequently detected in cases of neurological symptoms and infections (49%) (Table 3). EV-B (*n* = 20 infections, 95.2%) were found to result in the majority of neurological infections, with echovirus 30 (E-30) (*n* = 5, 23.8%) and echovirus 16 (*n* = 4, 19%) observed to have the highest frequency (Table S8). In contrast, no neurological infections from enteroviruses were found in adults or children with a transplant. Meanwhile, enteroviruses found in adults with a transplant were most commonly associated with either respiratory infections (four EV-D and two EV-C) or gastrointestinal infections (five EV-C and one EV-D) (Table S8). Gastrointestinal infections were also more likely (*P* < 0.05) to occur in adult transplant patients, compared to nontransplant adults. Interestingly, CV-A22 (EV-C) was found to be the causative agent in 3/6 gastrointestinal infections in adult transplant patients (Table S8).

### Severity associated with enterovirus infections.

Enterovirus infections have significant variations, not only in clinical manifestations, but also in disease prognosis. In order to determine if patients with a transplant had any potential differences in severity following an enterovirus infection, we examined length of hospital stay, symptom persistence and enterovirus recurrence in children (<16 years) and adults (Table 4).

**TABLE 4:** Clinical severity attributed to the detected enterovirus

Clinical severity factors*^1^^*a*^			Nontransplant recipients		Transplant recipients	
			Children (*n* = 74)	Adults (*n* = 43)	Children (*n* = 6)	Adults (*n* = 11)
Enterovirus infections	Length of hospital stay	Outpatient appointment	13 (17%)	13 (30%)	2 (29%)	9 (69%)
		2-6 days	42 (54%)	23 (53%)	2 (29%)	3 (23%)
		7-30 days	20 (26%)	7 (16%)	3 (43%)	1 (8%)
		>30 days*^2^	3 (4%)	0	0	0
	Recovery	Full recovery	67 (86%)	37 (86%)	4 (57%)	8 (62%)
		Persistence of symptoms	11 (14%)	5 (12%)	3 (43%)	5 (38%)
		Mortality	0	1*^3^ (2%)	0	0
		Total no.	78	43	7	13
Patients	Enterovirus recurrence*^4^	One infection episode	142 (88%)	62 (97%)	6 (50%)	21 (81%)
		Two infection episodes	18 (11%)	2 (3%)	4 (33%)	4 (15%)
		Three infection episodes	2 (1%)	0	2 (16%)	1 (4%)
		Total no.	162	64	12	26

a *1, Percentages were calculated from the total number of causative enterovirus infections for length of hospital stay and recovery. *2, One child without a transplant was still in hospital after the study was completed. *3, Coxsackievirus B5 myocarditis.*4, The percentage of enterovirus recurrence was calculated based on the total number of patients with enterovirus detections detailed in Tables 1 and 2.

**(i) Length of hospital stay.** The majority of enterovirus infections in children without a transplant required hospital admission (83%) (Table 4). A total of 54% (*n* = 42) of children without a transplant had a short-term stay of 2–6 days and 30% (*n* = 23) had a stay longer than 7 days. On average, these children were likely to have a hospital stay of 6.5 days, with infections most commonly caused by EV-B (*n* = 32, 49.2%) and EV-D (*n* = 22, 33.8%) (Table S9). Longer admission periods were strongly associated with neurological infections. Hospital stays of >30 days were reported from an enterovirus A71 infection causing meningitis and two EV-D68 infections, one causing acute flaccid myelitis (AFM) and the other causing pneumonia. In addition, 70% (*n* = 5) of infections in children with a transplant led to hospital admission of two or more days. In these five cases, enteroviruses were found to have caused gastrointestinal infections. Meanwhile, 50% (*n* = 5) of gastrointestinal infections in nontransplant children led to a hospital stay of two or more days. However, children with a transplant did not have a significantly longer length of hospital stay (LOS) than children without a transplant (*P* = 0.58).

The majority of adults without a transplant were also admitted to the hospital (70%) (*n* = 30) (Table 4) and tended to have a short-term hospital stay (average of 4.5 days), most commonly from neurological infections (*n* = 20, 66.7%). EV-D68 infections tended to be more severe, with six infections in children and three infections in adults resulting in a LOS of 7–30 days. Adults with a transplant were more likely to be seen in an outpatient setting with only 31% of enterovirus infections leading to hospital admission (average of 2.8 days). As there were only two group C infections in nontransplant adults, statistical power was not sufficient to determine differences in severity. However, it could be noted that three out of the four infections in adults with a transplant that caused a hospital stay of more than 2 days were caused by EV-C (Table S9). Overall, nontransplant patients tended to have a longer LOS compared to transplant patients, with an average of 5.2 days (most commonly from neurological infections) and 3.3 days (most commonly from gastroenteritis or respiratory infections), respectively (*P* = 0.014).

**(ii) Patient recovery.** Most patients with an enterovirus infection made a full recovery (82.3% infections) (Table 4). Overall transplant patients appeared more likely to have symptom persistence (*P* < 0.05). However, this was not found to be significant between children (*P* = 0.084) or adults (*P* = 0.104). Of note, adults with a transplant had symptom persistence from four EV-C infections: two cases of gastrointestinal symptoms from coxsackievirus A1 and two cases of respiratory symptoms from enterovirus C109 and EV-C105 (Table S10). An additional adult with a transplant had persistent respiratory symptoms from an EV-D68 infection (Table S10). Meanwhile, adults without a transplant had no symptom persistence from EV-C infections, but persistent neurological (*n* = 4, 66.6%) and respiratory (*n* = 1, 16.6%) symptoms, from EV-B and EV-D (Table S10). An additional adult had lingering symptoms from a coxsackievirus B5 infection causing myocarditis. Children without a transplant had symptom persistence following EV-A (*n* = 3 infections, 27.3%), EV-B (*n* = 2 infections, 18.2%), and EV-D (*n* = 6 infections [54.5%] including a child with AFM).

**(iii) Enterovirus recurrence.** Although the majority of patients in this study had only one infection episode, patients with a transplant were more likely to have a second and third enterovirus infection (Table 4). Indeed, 50% of children and 19% of adults with a transplant had more than one episode, compared with 12.3% and 3.1% of nontransplant children and adults, respectively (Table 4). This was found to be significantly different between children (*P* = 0.002) and adults (*P* = 0.020). Although multiple infection episodes were rare, five patients were found to have had three separate infections with the same/different enterovirus genotype. Of note, in the transplant population, one child had three separate E-11 infection episodes, while another had a CV-A4 infection followed by a co-enterovirus infection from EV-C104 (respiratory sample) and CV-A6 (plasma sample). Finally, an adult with a transplant had three separate CV-A22 infection episodes, highlighting the possibility for chronic enterovirus infections.

## DISCUSSION

Enterovirus infections can cause a diverse range of illnesses, even in healthy individuals (13–15). However, information detailing the clinical impact of enterovirus infections in a broad spectrum of transplant recipients is sparse, despite the high prevalence of these viruses in the community. With an aging population and increasing demand for transplants (16), it is important to investigate how infections could differ or compare to other patient populations and age groups. To our knowledge, this is the first study to characterize and describe all enterovirus infections in a diverse range of solid organ transplant patients.

Children (<16 years) without a transplant had the highest number of enterovirus detections, particularly in small children (0–5 years) (Table 1). This is not surprising as enterovirus infections are particularly common in children, especially newborns; with one study finding an incidence rate of 26–50 per 100,000 live births (17). We found prematurity to be the biggest risk factor for an enterovirus infection in small children, which has been found previously (17, 18) (Table 1). Interestingly, older children (6–15 years) had at least one underlying condition in 70% of cases. Meanwhile, in adults without a transplant, pulmonary difficulties, such as asthma and malignancy, were the most frequently observed co-morbidities. One study has indicated that up to 70% of asthma exacerbation is associated with viral infections, with the majority of cases caused by rhinoviruses and enteroviruses (19).

The population of children with transplants is small, as a result they represent the smallest group in this study. Enteroviruses were most frequently detected in liver transplants in small children presenting predominantly with gastrointestinal symptoms (*n* = 4, 80%). As our hospital performs approximately 30 pediatric liver transplants annually, it could account for the high number of liver transplants in this age group (Table 2). Conversely, in adults with a transplant, lung recipients had the highest number of enterovirus detections (Table 2). Again, this is not too surprising as these patients tend to be tested more for respiratory infections, even with mild symptoms, which might explain why enteroviruses were so frequently detected in respiratory samples.

Generally, the frequency of EV-A and EV-B detections, which include the majority of the coxsackieviruses and exclusively the echoviruses, declined with increasing age (Tables 1 and 2). This has been similarly observed in other studies, with EV-A and EV-B typically found in children (20, 21). In contrast, while EV-C detections tended to increase with age, EV-D detections were consistently found across all age groups in the nontransplant group and exclusively in adults in the transplant group. In this study, we found a relatively high number of EV-C detections in the transplanted adult population. Furthermore, CV-A22 comprised of 50% of the total EV-C detections in adults with a transplant, detected solely in fecal samples and associated with gastrointestinal and respiratory presentation. EV-C infections have not been specifically investigated in transplant recipients, although in the general population these infections have been sporadically associated with severe disease (22–24). Our study suggests that transplant recipients are more likely to experience illness associated with EV-C compared to nontransplanted individuals (*P* < 0.05). Additionally, transplanted individuals tended to have more symptom persistence resulting from EV-C infections.

The most detected enterovirus in this study was EV-D68 and was primarily associated with respiratory infections. We found EV-D68 to have caused the respiratory infection in 72.7% (*n* = 24) of detections in children and 67% (*n* = 10) of detections in adults presenting with respiratory illness, reiterating EV-D68 as a respiratory disease pathogen. However, in adults with a transplant, we found EV-D68 to have caused the respiratory infection in 40% (*n* = 4) of detections. Nevertheless, it must be noted that transplant recipients tended to have more co-detections and could account for the lower number of detections found to have solely caused the infection (Table S7). Overall, children had a more severe EV-D68 infection than adults in our study, with a higher number of hospital stays (*P* < 0.05).

Neurological infections caused by enteroviruses were only observed in nontransplanted adults and children in our study. Interestingly, the genotypes resulting in infection were different in children and adults. While coxsackievirus B5 resulted in the majority of neurological infections in children, echovirus 30 was predominantly found in adults, followed by echovirus 16. These genotypes are classically associated with neurological infections, with multiple clinical reports and *in vitro* studies reporting echovirus 30 particularly to be highly neurotropic (25–27). Neurological infections were observed to have a LOS of 2–6 days, underscoring the morbidity of neurological enterovirus infections. Remarkably, there appears to be an absence of neurological infections in transplant patients. Although the population of transplant recipients is small, based on these numbers it seems unlikely that transplant recipients have an increased sensitivity to enterovirus neurological infections.

There is an ongoing debate about enteroviruses as a cause for gastrointestinal illness (28). In immunocompromised individuals it is extremely important to distinguish true EV-gastrointestinal infections from bystanders, particularly as the same symptoms may also be caused by immunosuppressive medication or chemotherapy, for which the treatment would be therapy reduction (29). Enteroviruses are often asymptomatically present in feces (30). The distinction can often only be made on clinical grounds after excluding other possible causes. In this study, we determined the clinical relevance of enterovirus detections in feces based on clinician’s notes retrospectively. Indeed, while enteroviruses were most frequently detected in feces from children without a transplant (*n* = 86 detections), only 10 were determined to have caused the infection (11.6%). This suggests that while enteroviruses may cause gastrointestinal symptoms in nontransplant children, the vast majority of detections are not associated with clinical illness. In transplanted children enteroviruses were found to cause gastrointestinal infections in a third of cases. Although the number of patients in this category is small to draw a definite conclusion, it does appear that children with a transplant tend to experience more gastroenteritis associated with enteroviruses than their nontransplant counterparts. Furthermore, while we only detected seven enterovirus gastroenteritis cases, five required hospital admission, suggesting gastrointestinal infections can lead to considerable morbidity, which has been observed previously (31). However, as there were no significant differences in LOS between children, it suggests that once symptoms are serious enough to warrant admission, the severity is most likely similar in both groups.

Adults with a transplant were also more likely to have an enterovirus gastroenteritis infection compared to nontransplant adults (*P* < 0.05). Additionally, they were also more likely to have a single outpatient appointment at the time of sampling and a shorter LOS, compared to nontransplant patients. This is not surprising as transplant patients are more likely to have a higher number of check-up appointments and any new symptom will be treated with urgency with the patient being tested, regardless of hospital admission. Nevertheless, the longer recovery time and enterovirus recurrence could suggest patients could benefit from additional care. More follow-up studies monitoring the prognosis of enterovirus infections could provide additional information for clinicians for patient management, particularly as studies investigating gastroenteritis in transplant recipients are currently limited (32, 33).

This study has some limitations which need to be addressed. Although we retrospectively examined a major transplant center in the Netherlands over the course of 4 years, the small number of transplant recipients limited statistical analysis. Additionally, as enteroviruses are highly diverse (33 genotypes in this study alone), and are associated with various clinical manifestations, it can render definitive conclusions challenging, due to the multiple comparisons required. Furthermore, as this study was carried out in a tertiary hospital, the overrepresentation of patients with complex medical histories or severe illness could imply that the nontransplant population were not necessarily previously healthy or immunocompetent. A future study could include non-university hospitals and primary care to provide a clearer picture of the clinical burden of enterovirus associated diseases. Finally, it should be noted that the enteroviruses described in this study are reflective of the circulation in the Netherlands during this 4-year period, including three outbreaks of EV-D68. As a result, fluctuations in enterovirus prevalence are to be expected outside this time frame and region.

In spite of these limitations, it is possible to draw a few careful conclusions from this study. First, we have found that enteroviruses are not necessarily more severe in transplant patients. This is especially the case for neurological infections, which we did not detect in the transplant population. Second, it may be the case that some enterovirus genotypes are more pathogenic in transplant recipients. We showed that nearly all EV-C detections occurred in the transplanted population. In contrast, EV-A and EV-B infections were more likely to affect younger children, with the transplant population not appearing to be especially vulnerable. Third, although the question of enteroviruses being regarded as a true gastrointestinal pathogen is not entirely solved, according to the clinician’s opinion, enteroviruses were more commonly thought to cause gastrointestinal infections in transplant individuals.

## MATERIALS AND METHODS

### Setting.

The University Medical Center Groningen (UMCG) is the largest transplant center in the Netherlands. The UMCG performs approximately 200 kidney (adults only), 60 liver, 35 lung and 12 heart transplants annually. Approximately 3,000 transplant recipients are being followed-up. All patient information gathered during admission or outpatient visits were recorded and extracted from the electronic patient database system.

### Identification of enterovirus detections.

An enterovirus was initially detected through reverse transcriptase real-time PCR, followed by genotyping using Sanger sequencing by targeting the viral protein 1 gene (9). Sequences were analyzed with BioNumerics (v6.6) (Applied Maths NV). A study list was generated of all enterovirus positive samples between January 2014 and December 2018, regardless of whether an enterovirus genotype had been obtained. Samples which had not achieved a genotype were referred to as “untypeable.” These samples were used to determine overall prevalence.

### Identification of transplant patients.

Patients were included in the study if they had been admitted to the UMCG for an overnight stay (≥ one night) or had symptoms during an outpatient appointment. Only patients with enterovirus detection after transplantation were included. An enterovirus infection episode was defined as a single clinical period, including clinical symptoms and viral detection. Repeated samples that yielded the same genotype from the same patient within a 3-week period were removed (34). If a patient relapsed from an infection after clinical improvement, it was defined as a new episode. The clinical outcome of an enterovirus infection could be determined by collecting the following data: additional hospital admissions, outpatient appointments or check-up phone calls from the attending physician. Symptom persistence was defined as the continuation of complaints which prompted the sample collection that were subsequently attributed to an enterovirus. Once each study population had been established, patients with untypeable samples were excluded from further analysis (Fig. 1).

### Data collection.

A retrospective examination of all medical records from selected patients was performed. The following clinical information was collected: age, gender, comorbidities, enterovirus clinical presentation, final diagnosis on discharge, length of hospital stay co-detections (microorganisms detected at the same time as the enterovirus), recovery and frequency of all enterovirus detections between January 2014 and December 2018. The following sample information was collected from BioNumerics: enterovirus genotype, sample material and viral load. Samples were collected depending on the clinical symptoms of the patients and tested in our syndromic panels for respiratory, gastrointestinal and neurological infections. If an enterovirus was detected in more than one sample, only the material with the highest viral load, i.e., the lowest cycle threshold value was used for sequencing.

### Distinction between clinical presentation and infection.

To investigate whether enteroviruses found in patients were thought to be the cause of the illness, we examined both the recorded clinical symptoms (which prompted the sampling) and the final diagnosis of the patient following a clinical investigation. The subsequent documented diagnosis was used to assess if the treating physician considered the detected enterovirus as incidental, or a contributing cause of the illness (either as a causative agent or part of an infection caused by more than one microorganism). Only detections where an enterovirus was deemed to be the causative agent were counted as an infection, leaving out the detections which were judged to be incidental findings.

### Statistical analysis.

Qualitative and quantitative data were analyzed using IBM SPSS (v23) and compared using chi-square tests, multinomial regression analysis and Mann-Whitney U tests to determine significant differences between transplant and nontransplant patients (*P* < 0.05).

### Ethical statement.

This study was evaluated by the local UMCG Ethics Committee and a waiver “METc-2021/143” was obtained. All samples and patient data were pseudonymized before analysis according to local guidelines.

### Data availability.

The data that support the findings of this study are available from the corresponding author upon reasonable request.
