# Supplementary material for: Enterovirus Infections in Solid Organ Transplant Recipients: a Clinical Comparison from a Regional University Hospital in the Netherlands
Source: Microbiol Spectr. 2022 Feb 9;10(1):e02215-21. doi: 10.1128/spectrum.02215-21 (PMC8826731; doi:10.1128/spectrum.02215-21)
Supplement: SUPPLEMENTAL FILE 1 — Supplemental material. Download SPECTRUM02215-21_Supp_1_seq5.pdf, PDF file, 0.2 MB [file spectrum02215-21_supp_1_seq5.pdf]

Supplementary Material

Table S1: Overview of detected enterovirus genotypes

Genotype*1	Family group	Non-transplant recipients		Transplant recipients	
		Number	Total %	Number	Total %
CV-A1	C	3	1.3	3	6.4
CV-A2	A	6	2.5	2	4.3
CV-A4	A	16	6.8	3	6.4
CV-A5	A	4	1.7	0	0.0
CV-A6	A	22	9.3	3	6.4
CV-A9	B	7	3	0	0.0
CV-A10	A	8	3.4	0	0.0
CV-A11	C	1	0.4	0	0.0
CV-A14	A	1	0.4	0	0.0
CV-A16	A	5	2.1	1	2.1
CV-A19	C	0	0	1	2.1
CV-A22	C	1	0.4	9	19.1
CV-B1	B	1	0.4	0	0.0
CV-B2	B	3	1.3	0	0.0
CV-B3	B	5	2.1	0	0.0
CV-B4	B	6	2.5	1	2.1
CV-B5	B	15	6.4	0	0.0
E-3	B	1	0.4	0	0.0
E-5	B	0	0	1	2.1
E-6	B	9	3.8	0	0.0
E-7	B	0	0	1	2.1
E-9	B	4	1.7	0	0.0
E-11	B	10	4.2	3	6.4
E-16	B	11	4.7	0	0.0
E-18	B	6	2.5	0	0.0
E-25	B	5	2.1	0	0.0
E-30	B	10	4.2	0	0.0
E-33	B	1	0.4	0	0.0
EV-C104	C	4	1.7	1	2.1
EV-C105	C	10	4.2	2	4.3
EV-C109	C	1	0.4	3	6.4
EV-A71	A	3	1.3	2	4.3
EV-D68	D	57	24.2	11	23.4
Total		236	100	47	100.0

**1 Duplicate detections from the same infection were removed.*

Table S2: Detected genotypes in all sample materials

All detections	Patient	Sample type*3	Genotypes																																	Total
			CV-A1	CV-A10	CV-A11	CV-A14	CV-A16	CV-A19	CV-A2	CV-A22	CV-A4	CV-A5	CV-A6	CV-A9	CV-B1	CV-B2	CV-B3	CV-B4	CV-B5	E-11	E-16	E-18	E-25	E-3	E-30	E-33	E-5	E-6	E-7	E-9	EV-A71	EV-C104	EV-C105	EV-C109	EV-D68	
**Child (<16 years)**	**TX*1**	Fecal					1		2	1	3		1					1		3							1		1		1					15
		Respiratory																																1		1
		Other*4											1																							1
	**Non-TX*2**	Fecal		3			3		6		15	1	8	5		2	3	2	5	5	4	5	1	1	4			3		1	3	1			3	84
		Respiratory		3							1	3	6	1		1	2	2	4	3	2		2					1		1			5	1	36	74
		CSF*5					1											1	3	2	3	2	1		1			2		2						18
		Blister fluid											2																							2
		Other		1									1		1				1																	4
	Total		0	7	0	0	5	0	8	1	19	4	19	6	1	3	5	6	13	13	9	7	4	1	5	0	1	6	1	4	4	1	5	2	39	199
**Adult**	**TX**	Fecal	2					1		8																					1	1		1	2	16
		Respiratory	1																														2	2	10	15
		Blister fluid											1																							1
	**Non-TX**	Fecal	3		1	1	1			1									1		1		1												1	11
		Respiratory												1					1													3	5		18	28
		CSF																2	3		4	1			5	1		3								19
		Blister fluid		1			1						3																							5
		Other											2						1																	3
	Total		6	1	1	1	2	1	0	9	0	0	6	1	0	0	0	2	6	0	5	1	1	0	5	1	0	3	0	0	1	4	7	3	31	98

**1 TX; Transplant, *2 Non-TX; non transplant.*3 Duplicate sample types from the same infections were removed *4 other sample types consisted of plasma and heart tissue. *5 Cerebrospinal fluid.*

Abbreviations: CV-A1; Coxsackievirus A1, CV-A10: Coxsackievirus A10, CV-A16; Coxsackievirus A16, CV-A2; Coxsackievirus A2, CV-A22; Coxsackievirus A22, CV-A4; Coxsackievirus A4, CV-A5; Coxsackievirus A5, CV-A6; Coxsackievirus A6, CV-A9; Coxsackievirus A9, CV-B1; Coxsackievirus B1, CV-B3; Coxsackievirus B3, CV-B4; Coxsackievirus B4, CV-B5; Coxsackievirus B5, E-11; Echovirus 11, E-16; Echovirus 16, E-18; Echovirus 18, E-25; Echovirus 25, E-30; Echovirus 30, E-33; Echovirus 33, E-6; Echovirus 6, E-9; Echovirus 9, EV-A71; Enterovirus A71, EV-C104; Enterovirus C104, EV-C105; Enterovirus C105, EV-C109; Enterovirus C109, EV-D68; Enterovirus D68

Table S3: Detected genotypes in all sample materials for each solid-organ transplant.

Sample types*3	Genotype*2	Solid-organ transplant					
		Lung	Heart	Kidney	Liver	Multi organ*3	Total
Fecal	CV-A1	0	0	0	2	0	2
	CV-A16	0	0	0	1	0	1
	CV-A19	1	0	0	0	0	1
	CV-A2	0	0	0	2	0	2
	CV-A22	4	1	3	1	0	9
	CV-A4	0	0	1	2	0	3
	CV-A6	0	0	0	1	0	1
	CV-B4	0	0	0	1	0	1
	E-11	0	0	0	3	0	3
	E-5	0	0	0	1	0	1
	E-7	0	0	0	1	0	1
	EV-A71	0	0	1	1	0	2
	EV-C104	0	0	1	0	0	1
	EV-C109	1	0	0	0	0	1
	EV-D68	1	0	1	0	0	2
Respiratory	CV-A1	0	0	0	1	0	1
	EV-C105	2	0	0	0	0	2
	EV-C109	1	0	1	0	1	3
	EV-D68	7	1	1	0	1	10
Blister fluid	CV-A6	0	0	1	0	0	1
Other*1	CV-A6	0	0	1	0	0	1
Total		15	2	11	17	2	49

**1 other sample types consisted of plasma and heart tissue.*2 Duplicate detections from the same infection were removed, except if genotypes were found in more than one sample type (n=2) . *3 lung and liver transplant.*

Table S4: Detected genotypes in children and adults for each solid-organ transplant.

Solid-organ transplant	Genotype*1	Child	Adult	Total
Lung	CV-A19	0	1	1
	CV-A22	0	4	4
	EV-C105	0	2	2
	EV-C109	0	1	1
	EV-D68	0	7	7
Heart	CV-A22	0	1	1
	EV-D68	0	1	1
Kidney	CV-A22	0	3	3
	CV-A4	1	0	1
	CV-A6	1	1	2
	EV-A71	0	1	1
	EV-C104	0	1	1
	EV-C109	1	0	1
	EV-D68	0	2	2
Liver	CV-A1	0	3	3
	CV-A16	1	0	1
	CV-A2	2	0	2
	CV-A22	1	0	1
	CV-A4	2	0	2
	CV-A6	1	0	1
	CV-B4	1	0	1
	E-11	3	0	3
	E-5	1	0	1
	E-7	1	0	1
	EV-A71	1	0	1
Multi organ*2	EV-C109	0	1	1
	EV-D68	0	1	1
Total		17	30	47

**1 Duplicate detections from the same infection were removed. *2 lung and liver transplant.*

Table S5: Number of co-detections contributing to an infection along with an enterovirus

Co-detection	Non-transplant recipients		Transplant recipients	
	**Number**	**Total %**	**Number**	**Total %**
Acinetobacter pittii and Rhinovirus	1	2.13	0	0.00
Adenovirus	5	10.64	1	6.67
Adenovirus and Epstein-Barr virus	0	0.00	2	13.33
Adenovirus and norovirus	2	4.26	0	0.00
Adenovirus and rotavirus	2	4.26	0	0.00
Adenovirus and sapovirus	0	0.00	1	6.67
Adenovirus, CoV-OC43, HMPV and sapovirus	1	2.13	0	0.00
Adenovirus, norovirus and rhinovirus	1	2.13	0	0.00
Astrovirus and *Pseudomonas aeruginosa*	0	0.00	1	6.67
Bocavirus	1	2.13	1	6.67
Bocavirus and HPIV-1	1	2.13	0	0.00
CoV-Nl63 and rhinovirus	1	2.13	0	0.00
Epstein-Barr virus, rhinovirus and norovirus	0	0.00	1	6.67
Enterobacter cloacae complex and HPIV-3	1	2.13	0	0.00
Giardia lamblia	1	2.13	0	0.00
HMPV and *Staphylococcus aureus*	1	2.13	0	0.00
Norovirus	1	2.13	3	20.00
Norovirus and sapovirus	1	2.13	0	0.00
HPIV-2	2	4.26	0	0.00
HPIV-3	0	0.00	2	13.33
HPIV-1 and *Staphylococcus aureus*	1	2.13	0	0.00
HPIV-2, rhinovirus and *Staphylococcus aureus*	1	2.13	0	0.00
Rhinovirus	14	29.79	1	6.67
Rhinovirus and HPIV-4	1	2.13	0	0.00
Rhinovirus and *Pseudomonas aeruginosa*	1	2.13	1	6.67
Rhinovirus and HRSV B	1	2.13	0	0.00
Rhinovirus and *Staphylococcus aureus*	0	0.00	1	6.67
Rhinovirus and *Streptococcus pneumoniae*	1	2.13	0	0.00
HRSV A and B	1	2.13	0	0.00
HRSVB	2	4.26	0	0.00
Sapovirus	2	4.26	0	0.00
**Total**	**47**	**100**	**15**	**100**

Abbreviations: Human coronavirus OC43; CoV-OC43, *Human metapneumovirus;* HMPV, Human parainfluenza type 1; HPIV-1, Human coronavirus NL63; CoV-NL63, Human parainfluenza type 3; HPIV-3, Human parainfluenza type 2; HPIV-2; Human parainfluenza type 4; HPIV-4, Human respiratory syncytial virus; HRSV

Table S6: Number of alternative pathogens found to be the causative agent according to the attending clinician

**Co-detections (enterovirus identified as innocent bystander)**	**Non-transplant recipients**		**Transplant recipients**	
	**Number**	**Total %**	**Number**	**Total %**
Influenza A virus	1	5.56	0	0.00
Campylobacter jejuni	3	16.67	0	0.00
Clostridium difficile	0	0.00	1	16.67
Escherichia coli	2	11.11	2	33.33
Haemophilus influenzae	1	5.56	1	16.67
Klebsiella pneumoniae	1	5.56	0	0.00
Norovirus	1	5.56	0	0.00
HPIV-3	1	5.56	0	0.00
Pseudomonas aeruginosa and *Streptococcus pneumoniae*	1	5.56	0	0.00
Rhinovirus	0	0.00	1	16.67
HRSV B	1	5.56	0	0.00
Staphylococcus aureus	1	5.56	0	0.00
Staphylococcus aureus and Streptococcus group A	1	5.56	0	0.00
Staphylococcus aureus and Streptococcus pneumoniae	1	5.56	0	0.00
Stenotrophomonas maltophilia	0	0.00	1	16.67
Streptococcus pneumoniae	3	16.67	0	0.00
**Total**	**18**	**100**	**6**	**100**

Abbreviations: Human parainfluenza type 3; HPIV-3, Human respiratory syncytial virus; HRSV

Table S7: Enterovirus detections versus infections according to the treating clinician

	Non-transplant recipients		Transplant recipients	
	Children (n=172 detections)	Adults (n=64 detections)	Children (n=17 detections)	Adults (n=30 detections)
Causative infection	78 (45.3%)	43 (67.2%)	7 (41%)	13 (43.3%)
Co-infection*[1]	41 (23.8%)	6 (9.4%)	6 (35.3%)	9 (30%)
Innocent bystander*[2]	50 (29.1%)	14 (21.9%)	4 (23.5%)	7 (23.3%)
Missing	3 (1.7%)	1 (1.6%)	0	1 (3.3%)

**1 Enteroviruses were thought to have contributed (as part of a co-infection with another microorganism) to the illness. *2 An alternative pathogen or illness was identified as the cause of the patients' clinical symptoms.*

Table S8: Enterovirus genotypes and diagnosis on discharge

Enterovirus causative infections		Patient population	Enterovirus genotypes																										Total
			CV-A1	CV-A10	CV-A16	CV-A2	CV-A22	CV-A4	CV-A5	CV-A6	CV-A9	CV-B1	CV-B3	CV-B4	CV-B5	E-11	E-16	E-18	E-25	E-30	E-33	E-6	E-9	EV-A71	EV-C104	EV-C105	EV-C109	EV-D68	
**Child (<16 years)**	Respiratory	**TX*2**								1																	1		2
		**Non-TX*3**						1		1	1	1	1			1												24	30
	Gastrointestinal	**TX**				1	1	2								1													5
		**Non-TX**				1			1	1					1		1			1		1		1	1			1	10
	Neurological	**Non-TX**		1	1			1			1			1	5	3	4	3	1	1		2	2	1				1	28
	Other[*1]	**Non-TX**						1		5			2			1						1							10
	Total	**TX**				1	1	2		1						1											1		7
		**Non-TX**		1	1	1		3	1	7	2	1	3	1	6	5	5	3	1	2		4	2	2	1			26	78
		**Total**		1	1	2	1	5	1	8	2	1	3	1	6	6	5	3	1	2		4	2	2	1		1	26	85
**Adult**	Respiratory	**TX**																								1	1	4	6
		**Non-TX**																								1		10	11
	Gastrointestinal	**TX**	2				3																					1	6
		**Non-TX**			1												1									1			3
	Neurological	**Non-TX**									1			2	3		4	1		5	1	3						1	21
	Other[*1]	**TX**								1																			1
		**Non-TX**		1	1					5					1														8
	Total	**TX**	2				3			1																1	1	5	13
		**Non-TX**		1	2					5	1			2	4		5	1		5	1	3				2		11	43
		**Total**	2	1	2		3			6	1			2	4		5	1		5	1	3				3	1	16	56

**1 Hand Foot and Mouth disease, myocarditis, sepsis and fibril illness *2 TX; Transplant, *3 Non-TX; non transplant.* Abbreviations: CV-A1; Coxsackievirus A1, CV-A10: Coxsackievirus A10, CV-A16; Coxsackievirus A16, CV-A2; Coxsackievirus A2, CV-A22; Coxsackievirus A22, CV-A4; Coxsackievirus A4, CV-A5; Coxsackievirus A5, CV-A6; Coxsackievirus A6, CV-A9; Coxsackievirus A9, CV-B1; Coxsackievirus B1, CV-B3; Coxsackievirus B3, CV-B4; Coxsackievirus B4, CV-B5; Coxsackievirus B5, E-11; Echovirus 11, E-16; Echovirus 16, E-18; Echovirus 18, E-25; Echovirus 25, E-30; Echovirus 30, E-33; Echovirus 33, E-6; Echovirus 6, E-9; Echovirus 9, EV-A71; Enterovirus A71, EV-C104; Enterovirus C104, EV-C105; Enterovirus C105, EV-C109; Enterovirus C109, EV-D68; Enterovirus D68

Table S9: Enterovirus genotypes and length of hospital stay

Enterovirus causative infections		Patient population	Enterovirus Genotypes																										Total
			CV-A1	CV-A10	CV-A16	CV-A2	CV-A22	CV-A4	CV-A5	CV-A6	CV-A9	CV-B1	CV-B3	CV-B4	CV-B5	E-11	E-16	E-18	E-25	E-30	E-33	E-6	E-9	EV-A71	EV-C104	EV-C105	EV-C109	EV-D68	
**Child (<16 years)**	Outpatient appointment	**TX*1**								1																	1		2
		**Non-TX*2**				1		1		3					1					1		1			1			4	13
	2 to 6 days	**TX**						2																					2
		**Non-TX**		1	1			1	1	3	2		1	1	3	2	3	2	1	1		2	2	1				14	42
	7 to 30 days	**TX**				1	1									1													3
		**Non-TX**						1		1		1	2		2	3	2	1				1						6	20
	>30 days	**Non-TX*3**																						1				2	3
	Total	**TX**				1	1	2		1						1											1		7
		**Non-TX**		1	1	1		3	1	7	2	1	3	1	6	5	5	3	1	2		4	2	2	1			26	78
		**Total**		1	1	2	1	5	1	8	2	1	3	1	6	6	5	3	1	2		4	2	2	1		1	26	85
**Adult**	Outpatient appointment	**TX**	1				2			1																	1	4	9
		**Non-TX**		1	2					4											1					1		4	13
	2 to 6 days	**TX**	1				1																			1			3
		**Non-TX**								1				1	2		5	1		5		3				1		4	23
	7 to 30 days	**TX**																										1	1
		**Non-TX**									1			1	2													3	7
	Total	**TX**	2				3			1																1	1	5	13
		**Non-TX**		1	2					5	1			2	4		5	1		5	1	3				2		11	43
		**Total**	2	1	2		3			6	1			2	4		5	1		5	1	3				3	1	16	56

**1 TX; Transplant, *2 Non-TX; non transplant *3 One child without a transplant was still in hospital after the study was completed.* Abbreviations: CV-A1; Coxsackievirus A1, CV-A10: Coxsackievirus A10, CV-A16; Coxsackievirus A16, CV-A2; Coxsackievirus A2, CV-A22; Coxsackievirus A22, CV-A4; Coxsackievirus A4, CV-A5; Coxsackievirus A5, CV-A6; Coxsackievirus A6, CV-A9; Coxsackievirus A9, CV-B1; Coxsackievirus B1, CV-B3; Coxsackievirus B3, CV-B4; Coxsackievirus B4, CV-B5; Coxsackievirus B5, E-11; Echovirus 11, E-16; Echovirus 16, E-18; Echovirus 18, E-25; Echovirus 25, E-30; Echovirus 30, E-33; Echovirus 33, E-6; Echovirus 6, E-9; Echovirus 9, EV-A71; Enterovirus A71, EV-C104; Enterovirus C104, EV-C105; Enterovirus C105, EV-C109; Enterovirus C109, EV-D68; Enterovirus D68

Table S10: Enterovirus genotypes and recovery

Enterovirus causative infections		Patient population	Enterovirus Genotypes																										Total
			CV-A1	CV-A10	CV-A16	CV-A2	CV-A22	CV-A4	CV-A5	CV-A6	CV-A9	CV-B1	CV-B3	CV-B4	CV-B5	E-11	E-16	E-18	E-25	E-30	E-33	E-6	E-9	EV-A71	EV-C104	EV-C105	EV-C109	EV-D68	
**Child (<16 years)**	Full Recovery	**TX*1**				1	1	2																					4
		**Non-TX*2**			1			3	1	6	2	1	3	1	6	5	4	3	1	1		4	2	2	1			20	67
	Persistence of symptoms/ home care	**TX**								1						1											1		3
		**Non-TX**		1		1				1							1			1								6	11
	Total	**TX**				1	1	2		1						1											1		7
		**Non-TX**		1	1	1		3	1	7	2	1	3	1	6	5	5	3	1	2		4	2	2	1			26	78
		**Total**		1	1	2	1	5	1	8	2	1	3	1	6	6	5	3	1	2		4	2	2	1		1	26	85
**Adult**	Full Recovery	**TX**					3			1																		4	8
		**Non-TX**		1	2					5	1			1	3		4	1		5	1	2				2		9	37
	Persistence of symptoms/ home care	**TX**	2																							1	1	1	5
		**Non-TX**												1			1					1						2	5
	Mortality	**Non-TX**													1														1
	Total	**TX**	2				3			1																1	1	5	13
		**Non-TX**		1	2					5	1			2	4		5	1		5	1	3				2		11	43
		**Total**	2	1	2		3			6	1			2	4		5	1		5	1	3				3	1	16	56

**1 TX; Transplant, *2 Non-TX; non transplant.* Abbreviations: CV-A1; Coxsackievirus A1, CV-A10: Coxsackievirus A10, CV-A16; Coxsackievirus A16, CV-A2; Coxsackievirus A2, CV-A22; Coxsackievirus A22, CV-A4; Coxsackievirus A4, CV-A5; Coxsackievirus A5, CV-A6; Coxsackievirus A6, CV-A9; Coxsackievirus A9, CV-B1; Coxsackievirus B1, CV-B3; Coxsackievirus B3, CV-B4; Coxsackievirus B4, CV-B5; Coxsackievirus B5, E-11; Echovirus 11, E-16; Echovirus 16, E-18; Echovirus 18, E-25; Echovirus 25, E-30; Echovirus 30, E-33; Echovirus 33, E-6; Echovirus 6, E-9; Echovirus 9, EV-A71; Enterovirus A71, EV-C104; Enterovirus C104, EV-C105; Enterovirus C105, EV-C109; Enterovirus C109, EV-D68; Enterovirus D68
